# Thwarting Alzheimer’s Disease through Healthy Lifestyle Habits: Hope for the Future

**DOI:** 10.3390/neurolint15010013

**Published:** 2023-01-28

**Authors:** Vijaya Laxmi Govindugari, Sowmya Golla, S. Deepak Mohan Reddy, Alisha Chunduri, Lakshmayya S. V. Nunna, Jahanavi Madasu, Vishwanutha Shamshabad, Mounica Bandela, Vidyani Suryadevara

**Affiliations:** 1 Department of Biotechnology, Chaitanya Bharathi Institute of Technology, Hyderabad 500075, India; 2 Department of Bioengineering, University of Illinois, Chicago, IL 60612, USA; 3 Department of Radiology, Stanford University, Stanford, CA 94305, USA

**Keywords:** Alzheimer’s disease, cognitive decline, sleep, diet, exercise, alcohol, substance abuse, smoking, hearing loss

## Abstract

Alzheimer’s disease (AD) is a neurodegenerative disorder that slowly disintegrates memory and thinking skills. Age is known to be the major risk factor in AD, but there are several nonmodifiable and modifiable causes. The nonmodifiable risk factors such as family history, high cholesterol, head injuries, gender, pollution, and genetic aberrations are reported to expediate disease progression. The modifiable risk factors of AD that may help prevent or delay the onset of AD in liable people, which this review focuses on, includes lifestyle, diet, substance use, lack of physical and mental activity, social life, sleep, among other causes. We also discuss how mitigating underlying conditions such as hearing loss and cardiovascular complications could be beneficial in preventing cognitive decline. As the current medications can only treat the manifestations of AD and not the underlying process, healthy lifestyle choices associated with modifiable factors is the best alternative strategy to combat the disease.

## 1. Introduction

Alzheimer’s disease (AD) is a devastating neurodegenerative disorder affecting millions of aging populations worldwide and is possibly the third leading cause of death after heart disease and cancer [1]. AD is a progressive neurologic disorder that causes the brain to shrink (atrophy) and brain cells to die. During the very early stage of AD, toxic changes occur in the brain, including abnormal build-ups of proteins that form amyloid plaques and tau tangles [2]. The causes probably include a combination of genetic, environmental, and lifestyle factors. AD currently affects 5.8 million persons in the USA and is a common cause of dementia usually accompanied by other neuropathology.

Dementia manifests in four types: AD is characterized by the widespread deposits of tau tangles and amyloid plaques. Hyperphosphorylation of tau and accumulation of neurofibrillary tangles (NFTs) causes disintegration of the neuronal cytoskeleton and leads to progressive synaptic dysfunction, loss of dendritic spines, and neuronal death [3]. Various cellular stresses are known to result in upregulation of neuroprotective, neurotrophic amyloid precursor protein (APP), which when further processed leads to accumulation of toxic β-amyloid aggregates and thus neuroinflammation. Abnormal accumulation of Tau and TDP-43 proteins in the neurons of frontal and temporal lobes is seen in fronto temporal dementia [4]. In Lewy body dementia, the abnormal deposition of α-synuclein protein called “Lewy Bodies” affects the chemical messengers of the brain [5]. Vascular dementia is characterized by blood clots and disruption of blood flow to the brain [6].

AD is not a part of normal aging but likely to increase with age. Non-modifiable risk factors include: a family history with AD influence the chance of inheritance. The complex genetic factors, mutations in apolipoprotein E gene (APOE) [7], and variations in related genes are known to inherit the disease [8]. One extra copy of gene for amyloid protein production in down’s syndrome leads to the early occurrence of AD compared to normal people. 90% of the cases enlisted to date are found to be sporadic and the etiology of sporadic nature is still unknown, which implies genetics play a minor role in AD. The risk of AD was more likely to occur in females, one of the main reasons being their longevity of life span than men [9]. More devastating is that people with traumatic head injuries are more prone to AD within six months of injury [10]. The particulates of environmental pollution are known to speed up the process of neurodegeneration [11]. High blood pressure and high cholesterol levels are likely to increase the risk of AD [10]. Given the fact that there is no existing therapeutic intervention to treat AD, prevention of the disease is the best strategy. This review paper focuses on the modifiable risk factors of AD which include lifestyle, type of diet, substance use, alcohol, social life, sports, education, sleep etc.

## 2. Modifiable Risk Factors

### 2.1. Diet

Among lifestyle and environmental aspects which contribute to AD, diet is one of the primary risk factors and is crucial in AD pathology, as summarized in Table 1. Dietary factors are considered as the most compelling risk factors that headway the disease and have protective prospects in them [10]. The diets rich in saturated fatty acids, alcohol, carbohydrates (mainly fructose), and relatively lesser extent of dietary fats, cholesterol in diets may lead to AD’s progress [10]. Mediterranean diet, ketogenic diet, dukan diets, dietary approaches to stop hypertension (DASH) etc., are some of the neuroprotective diets rich in omega-3 fatty acids, antioxidants, polyphenols etc., [12]. The hallmark of this pathophysiology is represented by the advanced glycation end products of concerned diet components. The glycation led to a deficiency of cholesterol and fats in the brain which are elicitation components for synapses, signaling, and mitochondrial functioning [13]. This ultimately triggers oxidative stress, neuroinflammation, free radical production and induces mental retardation/dementia [14].

Several cohort studies have been investigated to correlate diet and AD. The probable causes of AD are known to result from higher animal fat intake, lower fiber content in diet, and increased total caloric and fat consumption [10]. Diets with high caloric consumption and fats are in greater intensity to produce free radicals, which in moderate amounts are useful for body processes and in higher amounts furtherance to more rapid aging [27]. Diets which include processed foods produce an acidic stomach which lead to the removal of bases such as alkali metals like magnesium, calcium, potassium etc., which are used to neutralize the acidic issues and higher adsorption of transition metals in the human system. Higher/lower adsorption of transition metals ions leads to leaching out of calcium ions from bones which attributes to AD [28]. Refined carbohydrates such as bread from white flour consist of phytate which reduces the adsorption of metal ions resembling higher proteins in diets [29]. Higher intake of essential fatty acids such as alpha-linolenic acid (ALA); n-6 polyunsaturated fatty acids(PUFA) are known to be immensely associated with onrush of AD. ALA (n-6 PUFA) increases the production of arachidonic acid, which in turn is a precursor for pro-inflammatory n-6 metabolites and enhances the production of cytokines and interleukin-1 [30].

Cholesterol, predominantly found in diet plan such as western diet with processed sugar, saturated fats, carbs, peanut, and vegetable oils, is one of the primary risk factors in the etiology of AD. While cholesterol has a role as an antioxidant, it also serves as a neural network scaffold, electrical insulator, and is utilized in synaptic delivery of neurotransmitters in the brain [31]. AD patients are examined with lower density lipoproteins [LDL] and levels of circulating cholesterol are largely affected by dietary plans. Dietary sugars and fats, especially saturated fats, aggravate AD. Elevated levels of cholesterol lead to amassing of hyperphosphorylated tau protein [32].

#### 2.1.1. Western Diet

Western diet [WD] is a term that defines the modern pattern of diet for western societies, which are highly incorporated with ultra-processed food, refined substances, fatty acids, high cholesterol etc., and composed of refined carbohydrates, saturated fats, salts, and lesser intake of vegetables, fruits, cereals, fibers, polyunsaturated and monounsaturated fatty acids, whole grains etc. WD adversely affects the functioning of gut microbiota, which ultimately reduces the absorption of nutrients from food. Gut microbiota dysbiosis accelerates low-grade inflammation followed by impairment of blood-brain barrier [BBB], synapse dysfunction, neuroinflammation, Aβ dyshomeostasis, which finally aggravates AD [33].

According to studies conducted on humans and animals, a high-fat diet results in a 50% decrease in bacteroides such as lactobacillus and bifidobacterium, which are prominent for the production of acetylcholine, neurotransmitters, γ-aminobutyric acids, etc., and a corresponding increase in proteobacterium, which alters the gut microbiota which eventually disrupts neurotransmission, synaptic loss, cognitive decline and ultimately AD [15]. Studies on WD reported on enhancement of AD pathological features in the brain, impair cognition, learning, and memory in humans [34].

High fat and high sugar diets (HFHS) reported to reduce the levels of brain proteins involved in synaptic plasticity, such as acetylcholine (Ach), dopamine, gephyrin, serotonin, synaptophysin (SYP), syntaxin-4, postsynaptic density protein 95 (PSD-95), and brain-derived neurotrophic factor (BDNF) [15]. HFHS intake increases the body weight and lipid parameters such as total cholesterol, HDL, LCL and enhances liver enzymes serum glutamate oxalate transaminase (SGOT), serum glutamate pyruvate transaminase (SGPT), and elevated Acetylcholinesterase (AcHE) levels in whole brain due to reduced cell density in hippocampus, oxidative stress, and free radical generation by cause of HFHS diet. A study investigating the impact of maternal environment and high-fat diet in early childhood released that were predicted to get transmitted through epigenetic mechanisms from mothers to offspring [35].

#### 2.1.2. Ketogenic Diet

Ketogenic diet [KD] is a nutrient plan with low carb, high fats, which brings the body to fasting and into a state of ketosis. KD systemically shifts the metabolism of glucose toward metabolism of fatty acids to trigger ketone bodies yielding [16]. This diet diminishes the generation of energy from glucose, accumulates Aβ plaques, and the formation of hyperphosphorylated tau protein. KD defects functioning of mitochondria, neuronal functions, triggers APP processing, results in neurotoxicity [16]. The modified ketogenic diet has neuroprotective actions which influence the neurons at different stages such as metabolic, signaling, and epigenetic levels [33]. KD with a high amount of acetone channels to hyperpolarize neurons and decreased neuronal excitability. It reduces the reactive oxygen species [ROS] generation, increases the activity of uncoupling proteins [UCP], and lessens after-effects of neurodegeneration [33].

#### 2.1.3. Mediterranean Diet

Mediterranean diet [MD] is a traditional dietary pattern consumed mostly in the borders of Mediterranean Sea, Italy, Spain, Greece. Humankind with high adherence to MD are proven to be exceptionally healthy and have reduced risk for incidence of AD and cognitive impairment [33]. MD is characterized by huge intake of fruits, vegetables, nuts, whole grains, seeds, heart healthy fats, moderate intake of fish, poultry, red and processed meat and alcohol, particularly red wine. Individuals with higher adherence to MD pattern are existentially less liable to AD.

Over the last few years, few studies attempted to investigate the association between Mediterranean diet and incidence of AD. The possible affiliation was first investigated by Scarmeas and colleagues in 2006 [36], comprising 2258 nondemented individuals who were potentially examined for 4 years. The 4 years follow-up study with varied distribution of adherence scores links up to lower the risk of AD by 40% in MD served individuals [21].

A diverse study was executed at German multicenter DELCODE (DZNE- longitudinal cognitive impairment and dementia study) in Germany, comprising 1.079 healthy individuals. A sample of 512 individuals was selected based on T1 weighted MRI and food frequency questionnaire, then were endowed for incidence of AD which included symptoms such as mild/subjective cognitive impairment. Distinct analyses were performed to disentangle the effects of Mediterranean diet on brain volume, memory functioning, deposition of amyloid plaques, and formation of Tau protein. Amid them, model-1 discusses interplay between MD, brain volume, and memory functioning, which proclaimed an indirect effect of MD on brain functions volume [37]. A longitudinal study in Scottish cohort over three years found that lower adherence to Mediterranean diet was found to be associated with reduction in brain volume [38]. Another model showed to untangle the moderation effect bred by MD on concurrent amyloid plaques and tau protein formation. These results laid out a positive relationship between MD, Tau protein formation, and brain gray matter. All these models exhibited a positive association between MD scores, brain volume, and its functioning in the hippocampus. These studies analyze liaisons between greater adherence to Mediterranean diet and lower risk of AD in aged individuals [39].

#### 2.1.4. Indian Diet

The Indian diet is enriched with diverse spices which includes curcumin, red chilies, coriander, Cuminum cyminum, cardamomum, Cinnamomum (cinnamon), pepper, ginger etc. Curcumin is a yellow curry spice that is widely used as an ingredient in the Indian diet due to its medical properties such as anti-inflammatory, antioxidant, anti-cancer, respiratory problems, infections etc. Curcumin prevents amyloid-β (Aβ) plaques deposition on receptors and has impotence to pass through the blood brain barrier, it ameliorates cognitive impairments and ameliorates synaptic functions [25]. Acetylcholinesterase, butyrylcholinesterase, and carboxyl esterase are typically found in AD patients, which can be treated using cardamomum, Cinnamomum, syzygium aroticum etc., which activates acetylcholine to inhibit AD pathogenesis. Saffron is found to be as effective as donepezil to treat mild AD. Pepper, zingiber, possess anti- inflammatory and antioxidant properties. All the spices in moderate amounts may aid in prevention/delay in onset of AD.

#### 2.1.5. Mediterranean—DASH Diet

The dietary approach to stop hypertension (DASH) is a non-pharmaceutical approach and is a entrusted diet plan for patients with hypertension, cardio-vascular disorders, and diabetes which routes to AD [40]. The primary components of DASH diets are high intake of fruits, vegetables, nuts, fish, whole grain, poultry and dairy products, low intake of red meat, saturated fatty acids, sweetened beverages, and sugars. Adherence to the DASH diet was found to be beneficial and may ameliorate cognitive functioning and reduce hypertension. The Encore study flaunted enhanced cognitive function in response to intake of DASH diet together with aerobic exercise [41].

The MIND is a dietary pattern developed to anticipate dementia and is a dietary combination of Mediterranean diet and DASH diet which are neuroprotective [42]. This pattern entails 10 brain healthy food elements which includes vegetables, leafy vegetables, berries, nuts, beans, whole grains, sea food, poultry, red wine, and olive oil. The unhealthy food elements contain red meat, butter, cheese, sweets, and fast food [43]. This pattern accents higher plant-based elements and limited intake of saturated fats and animals. The MIND diet recommends greater servings of food elements in the day-to-day diet comparatively in greater amounts than the Mediterranean diet and DASH diet [44,45,46]. A study published by Morris and his colleagues demonstrated the prevalence of MIND diet in reducing cognitive impairment and onset of AD unlike the other diet plans. The study findings imply, patients adhering to MIND diet were able to downgrade the risk of AD [43].

#### 2.1.6. Flexitarian Diet (or) Ketoflex 12/3 Diet

Ketoflex 12/3 is a plant food-based and mild ketogenic diet where ketone bodies are promoted by reducing carbs in diet and 12 h fasting window at least 3 h before sleep. This diet is mostly based on non-starchy plant products with high fiber and moderate amounts of meat [47]. Ketone bodies are alternative energy substrates for brain metabolism, reducing neuro-inflammation and increasing neuroprotective activities. Adherence to ketoflex 12/3 diet enhances brain functions and helps to reduce onset of AD [48].

#### 2.1.7. Vegetarian Diet

The vegetarian diet is one of the healthiest diet plans abstaining from meat products and it is popular owing to influences from religion, philosophy, ethics, the environment, culture, culinary, etc. The regime is mostly plant based which includes vegetables, fruits, legumes, nuts, whole grains with/without dairy products and minimizing intake of saturated and trans-fat which provide haler micronutrients to the brain [49]. The first Health study project conducted in 1974 [50] with devotees of Adventist church consuming vegetarian and meat diets were equated, which revealed results that devotees with vegetarian diets were less prone to dementia and delaying onset AD. Cohort studies conducted by Chicago health and Aging project and the Nurse’s health study, found that higher adherence to vegetarian diet was associated with declined cognitive impairment and delaying incidence of AD [26].

#### 2.1.8. Vitamins

Vitamins have potent antioxidant properties and greater nutritional components salient for brain functioning and to treat neurological disorders such as pellagra, beriberi, AD, PD, etc., [51], summarized in Table 2. Vitamin-E is a strong antioxidant that may aid vanquishing oxidative stress, Aβ aggregation, tau protein formation, and augmenting the cell signaling. Clinical studies were conducted to investigate the influence of ∝-tocopherol and γ-tocopherol on Aβ levels and neuronal cell death. Some researchers concluded that γ-tocopherol is important for neuroprotection whereas ∝-tocopherol was less effective in impeding the symptoms of AD [52]. A population-based study with geriatric patients concluded that participants with dietary intake of vitamin-E on an everyday basis resulted in lowering the risk of dementia [53]. Several studies found that high doses of vitamin-E is associated with increased mortality and heart failure. These findings aid in the attribute that adequate amounts of vitamin- E dosage may exhibit antioxidant effects on patients with mild AD [54].

Vitamin-A is a potent antioxidant that plays a key role in physiological function and converts into retinoic acid in pharmacokinetic events. Neuroinflammation and microglial activation are known causes of AD which results in varied concentrations of retinoic acid, where retinoic acid aids in inhibiting Aβ production [60]. Higher intake of carotenoid rich food was found to be associated with debilitating cognitive impairment [55]. It has been shown that the AD patients are deficient of vitamin-A, increasing the intake of β-carotene may aid in hindering the AD symptoms.

Vitamin-B known as water soluble vitamins are involved in various prominent biological activities such as DNA methylation, synthesis of monoamine-oxidase and phospholipids, etc. Homocysteine is non-protein amino acid generated in the methyl cycle, acts as a biomarker for vitamin-B deficiency and is associated with the formation of tau protein [56]. In a study, directed to examine the homocysteine levels in geriatric AD patients, where half of the individuals were in the placebo group, and the other half received vitamin supplements of B6, B12, and folic acid, which revealed a 30% decrease of homocysteine levels in the vitamin-B group [61]. Thus, adequate vitamin-B levels aid beneficial effects in AD patients.

Vitamin-D deficiency is common in older individuals due to lack of sun exposure, less intake of vitamin-D food and physical inactivity which lead to the risk of AD, PD, multiple sclerosis, etc., [57]. In an observational study of urban adults, it was revealed that a higher baseline of vitamin-D was associated with slower cognitive decline (Beydoun, A., et al., 2018). In an in CHIANTI study, which is a population-based study of aging, conducted with participants over 65 years of age in Chianti region of Italy flaunted that low levels of serum 25-hydroxyvitamin were linked to higher risk of cognitive decline. These findings suggest that vitamin-D deficiency might be an important risk factor for AD [62].

### 2.2. Exercise

AD is a disease caused due to the altercations in complex brain functions present in the regions of the brain such as the hippocampus and the neocortex. The onset of the symptoms of AD generally occurs in the older population [63]. The most common symptom that is observed is dementia or loss of memory. This gradually leads to loss in cognitive function as well as loss in behavioral functions as well. AD is known to primarily originate from the accumulation of senile plaques or the Aβ plaques which in turn leads to the accumulation of tau protein [64]. Aβ plaques are known to trigger cellular and molecular alterations thus causing progressive neurodegeneration in AD. Studies have shown that a decrease in accumulation of Aβ in the hippocampal and neocortex region has helped improving neuroprotection, cognitive function, memory, etc. Patients suffering from AD are also known to have low levels of brain-derived neurotrophic factor in their blood (BDNF) [65]. Physical exercise is believed to act as a contributor in the decrease of such accumulations due to production of antioxidative factors, improvement in synaptic plasticity, and production of degradation enzymes, as summarized in Table 3 and Table 4.

#### 2.2.1 Relation between Alzheimer’s Disease and Exercise

##### In Humans

Exercise was found to have a positive effect on patients with AD. Though it cannot be determined or concluded as a cure for AD, few experiments have proven a delay in the onset of symptoms of AD such as memory loss in people who exercise regularly [64]. Studies have shown that regular exercise has helped reduce dementia and helped increase concentration and memory. A study conducted on people who were not suffering with AD disease has depicted results showing that the risk of both dementia and AD can be decreased with regular exercise when compared to patients who are not physically active [73]. A meta-analysis study that included 16 studies on people, who were both male and female, came to a conclusion that the risk of AD can be reduced to an extent of 45% and dementia to an extent of 28% with regular physical exercise but the required amount of physical activity has not been determined [77]. Aβ accumulation is known to be one of the primary causes of AD. One study conducted based on participants enrolled in CHS-CS, showed a reduction in Aβ, specifically amyloid β1—42 in plasma amyloid samples collected (for assays) from 1998–1999 and 2002–2003. These results were also supported by ruling out the involvement of removal of Aβ by the kidneys [69]. A study conducted in Germany, where participants who were born between 1930 and 1932 were grouped as C30 and those born between 1950 and 1952 were grouped as C50. Physical activity that was tracked were cardio-respiratory and muscular endurance, muscle strength, body position, and flexibility. This 14-year study proved that there was a decreased risk of AD and mild cognitive impairment (MCI) in people who regularly exercise and perform better in neuropsychological tests [70,78]. Physical exercise has also shown to postpone or prevent cognitive decline and has benefited individuals in improving their memory, attention. A meta-analysis has also shown that physical exercise benefited people over the course of 12 months by improving their memory, processing speed through promotion of hippocampal neurogenesis [79].

##### In Mice

Experiments on mice have also shown a few promising results on the relation between physical exercise and AD. A study was performed on Male Swiss mice (10 mice per group, 21 ± 1 °C), where about 400 pmol/site amyloid β-_1-40_ was injected into the mice intracerebroventricularly. These mice were subjected to physical exercise, treadmilling, for 4 weeks for 5 days a week for 40 min each day. This experiment showed that physical activity contributed to the activation of the NLRP3 pathway which helped prevent any dysfunctions caused by amyloid β-_1-40_. This study also proved that physical activity prevented the increase of Iba-1 immunocontent in the hippocampal region caused due to inducing amyloid β-_1-40._ NLRP3 content increase was prevented due to physical training after the administration of amyloid β-_1-40_ [80]. Another study was conducted on the female and male Wistar rats bred in 22 ± 1 °C with 12-h light and dark cycles. Four weeks of involuntary exercise was chosen as the mode of physical exercise. The female mice were then mated with the male ones which were later injected with Aβ peptides. Intrauterine environment was studied after this injection and the cognitive function of the offspring was also tested. There was resistance to any metabolic changes that are usually triggered in offspring with mothers suffering from AD, especially those associated with Aβ accumulation [74]. Male transgenic mice, NSE/APPsw, grown in similar conditions were subjected to treadmill exercises for 3 weeks with the exercise duration varying each week. Transgenic mouse models have shown an increase in the levels of BDNF, IL-6 when subjected to regular exercise. Moreover, an increased expression in SIRT-1 has been observed to contribute to the decrease in Aβ content [73]. Exercises and physical activity have shown to reduce the accumulation of Aβ plaques. This could act as a potential preventive measure for AD in older patients. The amount of exercise to be done by people has not been measured, but studies have proven that regular exercise can help reduce the risk of occurrence of AD and improve cognitive function.

### 2.3. Education

Education plays a pivotal role in the trajectory of many humans’ lives. The period dedicated to formal education is associated with cognitive abilities in later stages. There are many studies that correlate education with dementia and AD. While there are studies that support the association that lower education leads to a higher risk of AD [81,82,83], there is also evidence that has identified that there is no significant correlation [84].

Dementia or memory loss in colloquial terms, disrupts the balance in a person’s daily basic and instrumental activities. The risk of dementia across various levels of education (low, middle, and high depending on the number of years of formal education) has been studied, with few studies pointing out the significant negative correlation between the level of education and risk of developing dementia. Although the categorization of education levels has been different in various research studies, the relation between less education and risk of dementia was found to be independent of this categorization. Among different causes of dementia, AD is the most prevalent form.

Few researchers suggest that highly educated people tend to perform well on psychometric tests when compared to the ones with low education, thereby leading to detection bias. Due to this, the possibility of a diagnosis of disease reduces, which further could affect the relation between high education and risk of AD [81]. But the concept of cognitive reserve in higher educated individuals is essential in mitigating the severity of symptoms of AD [85]. The cognitive reserve can be considered as a brain’s ability to withstand age-related damage, where higher cognitive reserve implies better ability to perform tasks. The cognitive reserve can be increased through education, and mentally stimulating activities [86] in the long term. Increasing cognitive reserve helps in healthy cognitive aging and better management of AD [87]. Education, though acting as a proxy of this reserve, has an unclear relationship with the aggregation of amyloid plaques and neurofibrillary tangles [87].

Cognitive reserve helps in lowering the burden of cognitive deterioration and might aid in delaying the onset of AD. But, once there are clinical signs, the rate of impairment in cognition (more specifically in executive functions) due to AD was found to be more rapid in the cases with higher levels of education [87]. Different studies reporting varying relations are a result of the heterogeneity of this disease. Therefore, education and related activities [86] aid in improvement of cognitive skills that have evidently helped in better management of cognitive decline in AD.

### 2.4. Social Life

AD is a progressive, persistent neurodegenerative disorder that causes a loss in cognitive and global functionality, leaving the patient reliant on others. As a consequence, quality of life (QOL) became a key metric to evaluate in people with AD, and even the efficacy of dementia intervention programs. Previous research has found that male patients have a better quality of life and have a smaller impact of the disease on their everyday lives than female patients, demonstrating the role of gender on QOL in AD patients (American Psychiatric Association [2013]). According to the literature, melancholy in AD patients causes a decline of ability to carry out activities of daily living (ADLs), culminating in a lower quality of life and accelerated cognitive deterioration [88]. As a result, psychological malaise has been linked to a higher risk of institutionalization and an increased likelihood and severity of AD [89].

Family structure and socialization are significantly impacted in AD patients, necessitating adjustment, reconstruction, and resilience strategies. Coping methods are critical among relatives of AD patients who have higher levels of anxiety and depression than families of patients with other chronic conditions. These approaches necessitate the blending of problems with daily tasks and peer interaction that have an impact on QOL [90]. Management techniques enable the AD patient to establish a consistent family and social life.

Evidently, Van der Mussele and team found that AD patients with depression had behavioral abnormalities, sleep difficulties, restlessness, and anxiety, proving that psychological morbidity or emotional discomfort predicted low QOL. A deterioration in ADLs, social interactions, and cognitive functioning has also been linked to psychological morbidity, stressing its negative impact on QOL [91]. High functioning and an active lifestyle, on the other hand, allow AD patients to maintain cognitive stimulation, which is essential for good QOL. Better cognitive performance and higher life satisfaction are linked to perceived social support and engagement in social activities, all of which contribute to improved QOL. Awareness of the condition and mindfulness were linked to a higher quality of life [92].

Early research has found that one’s social context, particularly social interactions, can have an impact on one’s behavior and mental health [93]. Socialization appears to play an important impact in general health as people age, according to research. Having to spend time with friends, neighbors, and family members can improve one’s quality of life, both physically and mentally. It is crucial to remember that people might feel alone even while they’re surrounded by their friends [94].

Finally, spirituality and religion are elements that can aid in the transition to a chronic disease and the patient’s overall well-being, and also serve as a way to cope, particularly for mildly affected individuals acting as the aid as a contemplative strategy in order to cope with the obstacles of cognitive decline and find a logical true essence to help them. It has indeed been linked to improved QOL, allowing patients to feel more secure and keep their degree of self [95]. Spirituality was discovered to be a mediator between numerous psychosocial factors, including stress, anxiety, melancholy, meaning and purpose, severity of the condition, and QOL (e.g., dementia, cancer, spinal cord injury, and multiple sclerosis) in patients with chronic illness. While functional deterioration in AD is cumulative, it is crucial to look into spirituality as a regulator of performance and quality of life in the initial diagnosis.

### 2.5. Substance Abuse

A substance may be defined as a psychoactive compound which has the potential to lead to health and social problems, including addiction. AD is a known irreversible neurodegenerative disease resulting in deterioration of cognition, behavior, function, and memory loss. Substance abuse during pregnancy, and in later life, leads to conditions promoting reduced brain growth [96]. Edinburgh research council conducted a study involving 87 brains of human subjects, including virus-free intravenous drug user brains, Alzheimer patients and controls. Tau positive hyperphosphorylated neuropil threads showed a significant increase in the frontal and temporal cortex of drug users, especially in the age group of above 30 years. In older-aged patients, it was observed that unexceptional amounts of drugs or alcohol use have adverse effects because of their significantly reduced ability to metabolize the substances [97], as summarized in Table 5.

#### 2.5.1. Cocaine

One of the pathological markers found in the AD brain is the presence of numerous neurofibrillary tangles mainly composed of hyperphosphorylated tau protein [98]. The imbalanced regulation of phosphorylation is involved in this pathological process but still is not clear what are the key kinases and/or phosphatases leading to tau hyperphosphorylation and its accumulation. From studies and reports, cyclin-dependent kinase 5 (CDK5) is one of the very few kinases found to be accumulated in AD neuronal cells. A study on rat model, resulted in the overexpression of CDK5 and its regulatory subunit p35 in the rat brain, when injected with cocaine. Cocaine, an enhancer of dopamine-mediated neurotransmission, in addition to activating CDK5, stimulates the activation of protein kinase A (PKA) via increasing the level of cAMP (cyclic—adenosine monophosphate) in neurons. The activated PKA, in turn, inhibits PP-1 (protein phosphatase) activity which could induce AD-like hyperphosphorylation of tau. Consequently, cocaine might induce an imbalance of multiple protein kinases and protein phosphatases and thus lead to hyperphosphorylation of tau and neurofilament. It is also suggested that enhancement of the dopaminergic system, such as seen in cocaine addiction, might be served as one of the causative factors for the imbalanced regulation in the phosphorylation system seen in AD brain and since hyper-phosphorylation of tau is considered as one of the early events in AD [98].

#### 2.5.2. Methamphetamine

Methamphetamine is a highly addictive amphetamine-type psychostimulant that acts on the central nervous system through multiple physiological pathways [115]. It is considered the second-highest illicit drug, with an estimated 0.4% annual global prevalence. Repeated use of methamphetamine may cause neurotoxicity with psychiatric symptoms, leading to Parkinson’s disease [116]. Several mechanisms proved to be potentially involved in neural damage (oxidative stress, mitochondrial dysfunction, and neuroinflammation), contributing to the neuronal degenerative patterns similar to AD. It was reported that meth exposure was involved in upregulating high mobility box protein 1 (HMGB1), consequently expressing higher levels of amyloid precursor protein (APP) [99]. Expression of APP was inhibited when HMGB1 was blocked in the pathway, indicating that HMGB1 may be a therapeutic target for reducing the accumulation of Aβ plaques in the brain and reducing the risk of AD progression caused by meth use. The studies reveal that neuroinflammation, which is common in AD brains, may act as a mediator for meth-induced APP expression through HMGB1 [99], shedding light on the potential mechanism by which meth exposure causes neurotoxicity and neurological deficits in AD. Although, it is significant to note that other studies have showed that meth in low-to-moderate doses can improve cognitive function and protect the brain. Acute low-dose meth use has improved working memory, severely impaired in AD. In the coming years, it will be crucial to determine the optimal pharmacology of meth as an anti-AD agent and the parameters necessary for a proper clinical application.

#### 2.5.3. Benzodiazepine

The use of benzodiazepine (BZD) and related psychoactive medications is linked closely to a mildly elevated risk of AD. Neuropsychiatric symptoms such as anxiety and insomnia are two prodromal or neuropsychiatric symptoms of dementia/AD that are treated with BZDs. In developed nations, the proportion of elderly persons using BZD ranges from 9 to 32%. Drowsiness, an increased incidence of falls and hip fractures, as well as mobility issues, are some of its negative side effects and episodes. Despite the advice, BZDs are frequently used over an extended period. Since AD accounts for 60–80% of dementia cases, it has been postulated that its prolonged use will speed cognitive decline and increase dementia risk [100]. 

### 2.6. Smoking

It has been several decades since the evidence for an association between smoking and AD has been reviewed. Intensive research has found that smoking is one of the modifiable factors which when altered affects the prevalence of the disease [117].

Tobacco smoke contains five thousand compounds of which nicotine is the most studied and is known to have a toxic effect on the brain [118]. According to research, this primary constituent of tobacco can cause or prevent AD depending on its use. From a study conducted by Brody [119], to find the effect of nicotine on brain activity using functional imaging, it was examined that short-term use of this improves cognitive function by activating the prefrontal cortex, thalamus, and visual system; and also increasing the dopamine concentration in the ventral striatum and nucleus accumbens, and long-term use of this is associated with reduction of availability of α₄β₂ nicotinic acetylcholine receptors (nAChR), which play a key role in synaptic transmission, in the thalamus and putamen and decrease monoamine oxidase A and B activity in basal ganglia [118]. Together with these findings, he concluded that smoking affects attentional performance, mood, anxiety, and irritability.

Nicotine is found to influence multiple cognitive domains which include memory, visual reasoning, visuospatial/constructional, and attention. Correspondingly Timothy and his associates reported that cigarette smoking is correlated to cortical thinning in those regions which are involved in early AD [120]. It has been reported that cigarette smoking hinders nitric oxide (NO) synthesis in the cerebral vascular endothelial cells leading to impaired cerebral blood supply in the brain and promoting the production of Aβ [121]. 

Additionally, other findings have obtained evidence that smoking-induced oxidative stress is the mechanism that is promoting AD neuropathology, wherein F2-isoprostane levels were used as markers of the oxidative stress mechanism. This oxidative stress caused by smoking is known to be involved in the initiation of amyloid deposition, which is observed in AD [122]. Free radicals present in cigarette smoke are oxidative stress-inducers and these radicals promote the production of Aβ through activation of JNK and PKR-eIF2α-signaling pathways [123]. Furthermore, neuroinflammation is another possible mechanism that links the neuropathology of AD and smoking.

Besides nicotine, tobacco consists of neurotoxic metals such as Al, As, Cd, Co, Cr, Cu, Hg, Mn, Ni, Pb, Se, Tl, V, and Zn at higher concentrations. Dyshomeostasis of these metals is reported to play a role in the pathogenesis of AD. For example, Cu is known to be linked with Aβ peptide, which is also involved in Aβ protein precursor [124]. Furthermore, in AD, high levels of Zn promote binding of Zn to β-amyloid which leads to neurodegeneration with the genesis of fibrillar Aβ aggregation [125]. Fe deposition in the brain promotes β-secretase to cleave Aβ precursor protein by negatively correlating with furin protein, thus increasing the Aβ concentration [126]. Mn is also proven to be involved in the pathogenesis of AD through the formation of senile plaques by dysregulation of manganese superoxide dismutase scavenger system [127].

However, few studies stated that nicotine in cigarettes may have some neuroprotective properties or may be associated with the decreased risk of AD but these studies were later proven wrong as there is no compelling evidence that supports nicotine protects against AD [128]. The reviewed evidence thus implicates nicotine as potentially harmful in the pathogenesis of AD and proven to be significantly associated with increased risk of AD. Thus, quitting smoking would be the best choice in lowering the risk factor for AD, as summarized in Table 5 [108,109].

### 2.7. Alcohol

Alcohol consumption is linked to many conditions with the pattern and amount of alcohol consumed determining the impact caused in these diseases [129]. Moderate consumption is often correlated with certain health benefits, although it is highly recommended to avoid consumption of any amount. In relation to AD, many studies suggest that a mild intake lowers the risk of AD, and a few studies describe the correlation as a “U shaped” pattern due to the lower AD risk through mild alcoholic intake when compared to non-drinkers and heavy drinkers [130]. Varying effects of the type of beverage (wine, beer, hard liquor) on AD have been studied. Some studies show that wine lowers the risk of AD due to the presence of polyphenols [131], when compared to hard liquor but controversial results have been obtained in other studies [132]. Hard liquor leads to faster progression in AD [133]. However, higher levels of wine can become neurotoxic.

There are only a limited number of studies correlating alcohol with the progression of AD. A study analyzed 360 AD patients in the early stage and heavy consumption of hard liquor has led to faster progression of AD. Alcohol may hamper the clearance of Aβ whose accumulation is a major hallmark in AD [134]. Hence, reducing consumption of alcohol can lead to improvement in the cognitive condition and thereby reducing progression of AD, as summarized in Table 5.

### 2.8. Sleep

Sleep is a biological process characterized by a decreased activity in various functions of the mind and body. This state is controlled by circadian rhythms whose function is essential for regulation of the sleep wake cycle. In a healthy condition, the circadian rhythms function to maintain the timing and occurrence of sleep depending on factors such as the surrounding light or darkness. Sleep can be broadly categorized into non-rapid eye movement sleep (non-REM) and rapid eye movement sleep (REM). There are multiple theories that correlate the necessity of sleep with functions such as memory consolidation, learning, cognitive development [135], body repair, and energy conservation, as summarized in Table 6.

Irrespective of the age group, an important lifestyle factor that is commonly overlooked is sleep. On average, a human spends about one-third of his life in sleep. Sleep is involved in the normal functioning of various processes of the human body. Disruptions in sleep in terms of quality and quantity are correlated with decline in cognitive abilities [141]. These disruptions are prevalent in about 25–66% of AD patients. During the onset of this disease or in the preclinical stage where symptoms are not discernible, along with the Aβ accumulation, there are sleep disturbances such as inefficient sleep patterns observed in many cases, yet often ignored.

Fluctuations in the sleep-wake cycle are linked to progression of AD. Aβ aggregation is the protagonist in AD, whose levels are linked with various factors [142]. Aβ levels fluctuate during sleep-wake cycles, and further increased Aβ production and reduced Aβ clearance were observed with disturbed sleep and increased wakefulness. Diurnal variations in the extracellular levels of Aβ can be found in interstitial fluid (ISF) and cerebrospinal fluid (CSF) of the brain. The amount of soluble Aβ is comparatively higher during the state of wakefulness than sleep. There is a bidirectional correlation between sleep-wake cycle and Aβ pathology [142]. While a dysregulated sleep-wake cycle can lead to Aβ formation and deposition, this deposition will in turn worsen the sleep patterns [142]. Deviation of the normal sleep requirements as in the case of acute sleep deprivation can lead to an increased production of this metabolite due to increase in the wakefulness time (or neural activity) during the night. Among various Aβ species, the most abundant one accounting for about 80–90% is Aβ40. The increased Aβ species in sleep deprivation conditions include Aβ38, Aβ40, and Aβ42. Along with the sleep-wake cycle, neuronal activity is also involved in regulation of the extracellular levels of soluble Aβ [143].

A major phase of life where sleep variations are common is the old age led by aging. From the studies incorporating the aging factor, it is evident how disrupted sleep and neurodegeneration are associated. Non-rapid eye movement, slow wave sleep (NREM SWS) declines with increase in human age [144]. NREM SWS is essentially known to play a role in memory consolidation. A study showed that a change in slow wave sleep can be observed with the amount of this phase reducing from 18.9% to 3.4% during the transition from 16–25 years to 36–50 years old. With the onset of old age, there is a major variation in the sleep patterns, with the deeper sleep stages being replaced with lighter ones, and recurring interruptions of sleep through frequent awakening, there is sleep fragmentation and poor quality of sleep [144]. These changes are closely linked with decline in brain activity in regions associated with sleep, memory, and cognitive abilities.

Another major hallmark is the tau protein, a microtubule-associated protein. During the state of wakefulness, this protein is released by active neurons in contrast to the state of sleep where it is cleared. The state of reduced sleeplessness or increased wakefulness leads to accumulation of hyperphosphorylated Tau forming neurofibrillary tangles, a characteristic of AD [142]. Orexinergic signaling is a component involved in the regulation of sleep-wake cycle, and its overexpression is reported to be a contributor in Aβ and tau aggregation, eventually leading to neurodegeneration [142].

A shift in brain health can be observed by changing the perspective from considering sleep as an interruption to activities (including late night studying and working) to giving it the importance as a contributor to good health. Few investigations have shown the impact of sleep deprivation and dementia developed after some years [145]. Although the effects of sleep deprivation might not always be immediate, it can eventually progress to cognitive decline, dementia, neurodegeneration, and an early death, as the age increases. Some measures such as following a sleep routine, avoiding caffeine [146] reduced exposure to electronic devices [147] can help increase the quality of sleep hence reducing the risk of dementia and hence AD in the long run. Research finds, insomnia in old age may aggravate cognitive decline allied with early-onset Alzheimer’s disease (AD) in adults without dementia and in adults with preclinical or prodromal AD [136]. According to the PSQI, there is a substantial correlation between CSF Aβ42 levels and sleep quality. In a study, it was discovered that patients with insomnia had considerably higher CSF Aβ42 levels [137]. The disturbance of metabolism in the brain caused by chronic sleep disorders may raise the chances of developing Alzheimer’s disease (AD) and other neurodegenerative illnesses. In patients with MCI, obstructive sleep apnea (OSA) is associated with higher levels of both T-Tau and P-tau in CSF suggesting that could be related to the pathophysiological processes involved in Alzheimer’s disease [138]. A study suggests that sleep disruptions are a sign of increased risk for AD which potentially complement increased Aβ levels, associated with fragmented SWS, suggesting that disrupted sleep may be an early sign of AD [139]. A study raises the possibility of using bright light treatment to improve circadian rhythm. The group of patients who were mildly demented showed remarkable improvement in circadian rhythm and cognitive performance, whereas the moderately and severely demented patients did not [140].

## 3. Improving Existing Conditions

### 3.1. Vascular Health

Vascular risk factors play a major role in arbitrating AD, these risks are strokes, hypertension, diabetes, heart failure, atrial fibrillation, homocysteine, smoking etc., which trigger cerebrovascular disorders and AD [148]. Stroke causes loss of neuronal tissues which might heighten amyloid plaques and tau proteins assemblage in the brain. Heart failure, atrial fibrillation causes hyper perfusion of brain which leads to nerve damage and hypoxia. Furthermore, increased speculations of emboli, lacunae, and white matter lesions in turn communed to AD [149]. Diet plans such as western diet embrace high density level [HDL] cholesterol, saturated fatty acids, high sugar, and fats are major determinants of heart dysfunctions. Omega-3 polyunsaturated fatty acids have appealed to be protective against heart diseases, it includes fresh fish, olive oil etc. polyphenol from fruits, vegetables, nuts, seeds, grapes wine, alcoholic beverages, heart healthy fat as such in the Mediterranean diet avert heart diseases. The Mediterranean diet is the best diet with more evidence to obviate vascular disease, AD, and even other chronic disorders [150,151].

### 3.2. Cardiovascular Health

Researchers are increasingly discovering a link between what is beneficial for the heart and the brain. The function and health of brain cells are dependent on adequate blood circulation to the grey matter. About a quarter of your blood is supplied to your brain with each heartbeat, carrying the essential carbs, fats, vitamins, hormones, and amino acids to provide your brain the energy it needs to recall information and think properly. There has been accumulating evidence of a strong link connecting AD and cardiovascular illness in recent years, notably in the areas of the heart disease’s tendency to deprive the brain of blood [152]. According to their preliminary findings substantially regulating high blood pressure can reduce the likelihood of moderate cognitive deficits, which is frequently a precursor to dementia. Proper and balanced living strategies, such as those of maintaining a healthy diet, getting regular exercise, not smoking, avoiding stress, and continually checking blood pressure and sugar levels, will help keep your heart in shape and reduce blood pressure, which can help prevent AD [153].

### 3.3. Hearing Loss

Age-related hearing loss is a common condition linked to ageing individuals. Deficits in both peripheral hearing and central auditory processing can contribute to this condition [154]. Suffering from hearing impairment affects the individual’s communication and auditory abilities. Due to lack of or reduced social interaction various behavioral and psychological symptoms arise. It was deduced that an impact on hearing ability has shown association with symptoms of dementia and AD incidence [155]. Epidemiological studies found that the hearing-impaired population reflected a higher risk of progression in cognitive decline [156]. A multicenter clinical trial done on the effect of hearing aids, fitted to patients suffering from both hearing impairment and AD did not show signs of improvement in QoL nor the neuropsychiatric symptoms, owing to the neurodegenerative disease’s stage of progression [155]. On the other hand, it cannot be ruled out to have a positive rehabilitative effect in younger patients and on the progression of early AD pathology. Use of hearing aids among patients with hearing loss was associated with prevention or delay in onset of AD along with other common age-related conditions such as dementia and depression [157]. It is imperative that timely diagnosis of hearing loss and the use of hearing aids can certainly improve the QoL and might redact the risk of AD and cognitive decline. Since recent studies found a correlation between hearing loss and alterations in brain physiology in animal models, an alliance with changes in molecular pathways can be associated with AD pathology and help improve the daily life of the patients [158].

## 4. Conclusions

Although there is significant progress in the pathological approaches for treating AD, they failed to show better efficacy than the current management of AD. While few drugs are currently used to treat AD directly, their efficacy in delaying disease onset is limited. Additionally, many of the treatment strategies developed are associated with major obstacles and historical failure. The challenges include the multifactorial complexity of the AD pathology, which leads to difficulty in developing a drug, and the blood–brain barrier (BBB), which is impermeable to available small molecule drugs. Since the current pharmaceutical treatments fail to cure this disorder, it is always better to prevent such diseases by alternative strategies which are based on lifestyle choices. Hence promoting a healthy lifestyle is a fundamental principle to prevent such diseases, since there is evidence that specific lifestyle and environmental aspects lower the risk of AD. Studies in elderly people has been shown that modifying vascular and lifestyle related risk factors improved the quality of life of patients by improving their cognitive reserve and reduce the risk of AD [159].

The modifiable risk factors that have been corner stoned for this review are diet, exercise, smoking, social life, substances used, sleep, alcohol consumption, and education. Improving hearing loss, heart health has also been found to be beneficial in preventing AD. There has been increasing evidence over the past few decades that describe the association between these modifiable factors and AD. A low carb balanced diet and low cholesterol, high levels of HDL or a Mediterranean diet has been reported to help delay or prevent the onset of AD symptoms. With regular exercise, the risk of both dementia and AD reported to be decreased due to reduced accumulation of Amyloid β plaques. Mental and physical challenges, spirituality, family structure, and often social interactions significantly impact an AD patient’s overall well-being. The brain is known to develop more internal connections through these challenges, which protect against dementia. Smoking, using intoxicating substance abuse, hard liquor, fluctuations in the sleep-wake cycle for a substantial period may bring upon dementia or accelerate the neuronal damage associated with AD. Inculcating a healthy lifestyle since early stages have a profound impact on lowering the risk of AD and when compared to the current pharmaceutical treatment strategies these are easy to implement, cost-effective, and usually safe. Formulating diet, exercise, social interactions specific to the communities around the world and disseminating the information to the public in the most comprehendible way will make it easy for them to incorporate it into their lives. Raising awareness among the communities around the world about the benefits of multidomain approaches in cultivating good lifestyle habits from early on in life will help prevent and mitigate the risk of AD.

## Figures and Tables

**Table 1:** Dietary plans and its efficiency.

Diet	Variables Included	Food Items Enlisted in Diet	Results	Ref
Western diet	Age, sex, education, adherence to diet plans	Saturated fatty acids, refined carbohydrates, refined grains, high fat dairy products and sugars.	Adherence to western diet is associated with more cognitive decline and onset of AD.	[15]
Flexitarian diet (or) Ketoflex 12/3 diet	Age, sex, reducing insulin resistance.	High quality fish, meat, cooked and uncooked vegetables, fruits, nuts, avocado, olive oil, no/less gluten, and dairy products	Ketoflex diet achieved blood chemistry and ketosis which are required for anticipation of AD	[16]
Ketogenic diet	Age, sex, education, APOE proteins, cardiovascular risk factors. Age, sex, food habits	Saturated and trans fatty acids, 70% fats, 20% proteins, <10% carbohydrates	risk of cognitive problems Adherence to KD reduced symptoms of AD	[17,18]
Dietary approaches to stop hypertension (DASH) (or) Mediterranean—DASH diet (MIND)	Age, sex, physical activity, diabetes, strokes, obesity, education, low BMI, hypertension	High intake of vegetables, fruits, nuts, whole grains, low fat dairy products,	The DASH diet can lessen the symptoms of AD and MIND diet is a better safeguard against AD.	[19,20,21]
Mediterranean diet	Age, sex, caloric intake, smoking, ethnicity, BMI index, exercise, comorbidity index, hypertension, diabetes, heart disorders, diet, and cognitive assessment.	Whole grains, fruits, vegetables, seeds, nuts, omega-3 polyunsaturated fats like olive oil, fish and moderate intake alcohol and red wine	Higher adherence to MD is associated with reduced risk of AD	[22,23,24]
Indian diet	Age, sex, food habits	Daily diet which entails turmeric, garlic, zingiber, cinnamon, pepper, cardamom, saffron, clove, cumin etc.,	Dietary supplementation with these spices aid in prevention and delay of onset AD.	[20,25]
Vegetarian diet	Age, sex, locality, vegan, ovo-lacto-vegetarian, education, smoking, drinking, marriage, and exercise.	Vegetables, fruits, cereals grains, seeds, nuts, mushrooms, including/excluding dairy products	Adherence to vegetarian diet is associated with reduced risk of AD	[26]

**Table 2:** Vitamins and AD associated results.

Diet	Variables Included	Food Items Enlisted in Diet	Results	Ref
Vitamin-A	Age, sex, dietary pattern, subjective cognitive function (SCF) assessment	Leafy vegetables, cereals, dairy products, sand foods rich in β-carotenoids	Ameliorates cognitive function, inhibits Aβ aggregation	[55]
Vitamin-B: vitamin-B6 and B12, folic acid	Age, sex, education, drinking and smoking habits, drug use, marital status, and medical history	Leafy vegetables, fruits, peas, meat, fish, dairy products	Suppresses the homocysteine level and oxidative damage, concealing cognitive decline	[56]
Vitamin-D	Age, lifestyle, medical history, drug use, and anthropometric data	Fish, cod liver oil, beef liver, eggs, fortified cereals, dairy, and plant milk products	Reduces neuroinflammation, mitigates Aβ plaques, regulates Calcium homeostasis, and ameliorates cognition in mild AD patients	[57]
Vitamin-E: Tocopherol and Tocotrienol	Age, mild AD patients, vitamin-E serum levels, cognitive performance	Vegetable oils, nuts, seeds, avocado and food rich in unsaturated fatty acids	Dietary supplementation with these spices aid in prevention and delay of onset AD.	[58,59]

**Table 3:** Exercise and Alzheimer’s disease in humans.

Number of Patients/Volunteers and Age Group	Suffering From	Variables Included	Exercises Performed	Time Period	Observations	Ref
Number: 200 Age: 50–90	Mild Alzheimer’s	Included MSME score > 19, age, Excluded presence of cardiac disease, severe psychiatric disease, alcohol abuse, participants with regular physical activity	Aerobic: Moderate-to-High Intensity	16 weeks (4 weeks—strength building, 12 weeks—aerobics)	No benefits on cognitive performance but improved neuropsychiatric symptoms	[63,66]
Number: 100 Age: 55–86	Mild Cognitive Impairment	N/A	Aerobic and Resistance Training	6 months of PRT and 18 of combined CT and PRT	6 months of PRT and Aerobics improved memory, attention, and executive functions	[64,67]
Number: 295 Age:—(Born between 1900–1920)	Decreased cognitive function. (MMSE score > 18)	MMSE score > 18	Walking and Games (including billiards, volleyball gymnastics, swimming)	Approximately more than 60 min a day.	Improved cognitive functions	[68,69]
Number: 381 Age: 74 (avg.)	Mild Cognitive Impairment and Alzheimer’s	MSME score, Subtests such as Memory, Visuospatial functioning, Verbal comprehension, Abstract thinking, Speed, Attention Neurological, mobility and Parkinson’s disease excluded. Dropouts also excluded	Physical fitness including exercises for muscle strength & endurance, flexibility, cardio—respirator	12-year follow up	Improved neuropsychiatric symptoms	[70]
Number: 153 Age: 55–93	Alzheimer’s Disease	MSME score 16.8, suffering from dementia for an average of 4.3 years	Aerobic, Strength training, balance, and flexibility	30 min a day—24 months	Increased physical health and function, decrease in depression rates	[71]
Number: 134 Age: 62–103	Mild to severe Alzheimer’s Disease	MMSE score, behavior changes, physical performance scores Age, sex, current medication, cholinesterase inhibitors, psychotropic treatments	Aerobic (including strength, flexibility, and balance training)	1 h, twice a week with a gap of at least 2 days for 12 months	Slower decline in ADL in patients following continuous exercise	[72]

**Table 4:** Exercise and Alzheimer’s disease in rodents.

Species	Growth Conditions	Variables Included	Type of Exercise	Observations	Ref
Transgenic Mice, NSE/APPsw Male	12: 12-h light and dark cycle Temperature of 23 ± 1 °C Humidity—50%	Mice expressing human APP mutant under NSE and maintained in genetic background of C57BL/6 X DBA/2 mice	Treadmill exercise First 2 weeks: 30 min/day—5 days Next 2 weeks: 50 min/day—5 days Next 3 weeks: 60 min/day—5 days	Reduced Aβ levels, improved spatial learning and memory, reduced Aβ-induced cell apoptosis	[73]
Wistar rats—*Rattus norvegicus albinus* Female—220–260 Male—300–350	12: 12-h light and dark cycle Temperature maintained at 22 ± 1 °C. Humidity: 50–60% Female rats subjected to pregnancy and induced with AβO	N/A	Swimming—5 days/week for 30 min each day.	Infants born with lesser cognitive defects. Improved brain metabolism of offsprings	[74]
Transgenic Mice, Tg-NSE/hPS2m	12: 12-h light and dark cycle Temperature of 22 ± 2 °C Humidity—50%	Mice expressing human PS2 mutant under NSE and maintained in genetic background of C57BL/6 X DBA/2 mice	Treadmill exercise Pre-exercise: 10 min/day—5 days 60 min/day for 5 days	Reduced Aβ-42 deposition, reduced tau phosphorylation levels	[75]
3xTg-AD mice Male and Female	12: 12-h light and dark cycle Temperature of 22 ± 2 °C	Mice possessing familial AD mutations PS1/M146V, AβPPSwe, tauP301L	Running wheel One month—ending at 4 months of age One/six months—ending at 7 months of age Six months—ending at 7 months of age	Improved muscle strength and coordination, improved exploratory behavior, reduced anxiety levels	[76]

**Table 5:** Table for substance abuse: drugs, smoking, and alcohol.

Substance Abuse	Age	% Or Duration of Intake	Effects of Substance Abuse	Ref.
**Drugs**				
Cocaine	Adults (15–64)	Cocaine: 3.5 mil. 1.2%	Induce hyperphosphorylation of tau proteins due to inhibition of PP1 on overexpression of CDK5	[98]
	Young adults (15–34)	Cocaine: 2.2 mil. 2.1% Worldwide *2021		
Methamphetamine	Adults (15–64)	Methamphetamine: 2.0 mil. 0.7% Worldwide *2021	Increased production of APP due to unregulated HMGB1 expression, resulting in accumulation of Amyloid β plaques	[99]
	Young adults (15–34)	Methamphetamine: 1.4 mil. 1.4% Worldwide *2021		
Benzodiazepine	Adults (50–64)	Benzodiazepine 30.6 mil. 12.9% (USA)*2019	Predisposition or onset due to GABAA-benzodiazepine chloride ionophore activity in susceptible individuals.	[100]
**Smoking**				
	Mean age of 81 years	Never, Ever, Continuing	Persistent smoking increased the onset rate of dementia	[101]
	60 years	Never and Current	Smoking amount and status have been associated with dementia and AD.	[102]
	32–87 years	Ever	Smoking was associated with increased risk of AD.	[103]
	Mean age 76.2 years	Current, never	Current smoking was the strongest risk factor associated with an increased risk of AD.	[104]
	≥65 years	Current	In comparison with never smokers, current smokers are more likely to develop AD	[105]
	≥55 years	Never, Past, Current	Current smoking has increased the risk of AD in persons without APOE ε4 allele.	[106]
	65–79 years.	Mid-life smokers	Smoking in midlife was shown to increase the risk of dementia, and AD. This association was limited to APOE ε4 carriers.	[107]
	43 to 70 years	persistent nonsmoker, ex-smoker, persistent smoker, recent quitter	Interventions to prevent or stop people from smoking may postpone cognitive decline in middle-aged persons	[108]
	≥60 years	continual smokers, short-term (less than 4 years) quitters, long-term (4 years or more) quitters, and never smokers	Smoking was associated with increased risk of dementia and long-term quitters had a reduced risk of dementia.	[109]
**Alcohol**				
	Mean age = 77.49 years	Age, sex, habit of alcohol consumption, genetics/ancestry, dependence symptoms due to alcohol consumption	The correlation of alcohol consumption and alcohol dependence was found with earlier and delayed AD Age of Onset Survival, respectively.	[110]
	Age = 40–59 years	Age at baseline, sex, drinking status, smoking status, total and HDL cholesterol, systolic BP, and BP medication status.	The correlation between alcohol consumption and change of brain volume was found to be non-significant.	[111]
	Mean age = 60.0 ± 11.1 years	Age, sex, duration illness year and drugs	Abstinence was found to be useful in slowing cognitive deterioration in AD patients who had a history of binge drinking.	[112]
	Mean age = 58.1 ± 8.3 years	Type of alcohol, alcohol intake dose, ethnicity, study design and sex	Drinkers had a decreased risk of AD than non-drinkers, with wine observed to lower its risk, furthermore. A non-linear and insignificant relation was observed between the alcohol dose and risk of AD.	[113]
	Age = 76–80 years	Age, sex, APOE E4 carrier status, Mild Cognitive Impairment at baseline, and alcohol consumption	Both total abstinence and over-drinking were linked to decreased cognitive performance.	[114]

**Table 6:** Sleep disruptions and AD-associated results.

Sleep Disruption	Sample	Variables Included	AD Associated Results	Ref
Insomnia	Mean age = 73 years, 385 cases 46–67 years, 23 cases	Age, gender, education, APOE E4 status, clinical diagnosis, number of prior exposures to cognitive test, sleep medication use, hypertension, diabetes, hyperlipidemia, stroke history, hearing loss, depression, anxiety, coronary heart disease, and current smoking status. Age, sex, educational level, occupation, CSF levels of Aβ and tau	Insomnia in non-demented elders was found to influence the correlation between cognitive decline and Aβ. Greater levels of Aβ42 were observed in insomnia patients, which was found to increase with duration of the condition.	[136,137]
Obstructive Sleep Apnea (OSA)	Mean age = 66.19 years, 57 MCI (mild cognitive impairment) cases	Age, sex, body mass index, sleep medication, smoking, hypertension, and heart disease	Cases with severe OSA were observed to have higher phosphorylated tau and total tau levels.	[138]
Changes in Slow Wave Sleep (SWS)	Mean age = 69.8 ± 6.4 years, 21 cases	Age, gender, education, sleep, Plasma Aβ values, and cortical thickness	Significant correlation between disturbed SWS and Aβ42, and shorter rapid eye movement (REM) sleep and reduced thickness in certain AD associated brain regions was observed.	[139]
Changes in circadian rhythm or sleep-wake cycle	Mean age = 79.9 years, 27 cases	Age, gender, any physical problems in past/present and sleep timings	Circadian rhythms and cognition had improved through bright light exposure, without any changes in AD-associated dementia. 3	[140]

## Data Availability

Not available.
